# Translations

**Published:** 1884-03

**Authors:** 


### Translations from Foreign Exchanges.

BY O. STROINSKI, M.D., CHICAGO.

### NEW OBSERVATIONS ON DECAPITATION.

Frishjof Holmgren, a Swedish physician, has reported to the Medical Society of Ujosala his recent observations on decapitation. In the first execution, the eyes of the decapitated were widely opened and the pupils contracted, three seconds after the blow ; twenty seconds after decapitation they commenced to dilate, and dilation was complete two minutes afterwards, after which the eyes turned to the right and upwards twenty-five seconds later. The reflex movements commenced forty-four seconds after decapitation, by slight twitchings of the muscles of the neck, and there was then a violent contraction of these muscles. The mouth was thrown downwards and to the left side ; the tongue seemed also to be turned to the left, and a few seconds afterwards the mouth, which had been widely opened, closed slowly. After a few slight contractions of the muscles of the face, a complete rest ensued one minute and forty-four seconds after decapitation. From the surface of the dissected neck the blood streamed one meter, and thirty-five seconds afterwards the blood flowed in cascades. On the body no movement was noticed. In the second execution, the author was placed in front of the culprit during decapitation. The flow of blood was thrown out in a jet of 1 m. 33 c.m. The author concludes that in decapitation the sensibility disappears instantaneously, and that decapitation is entirely painless.—*Hospitals Tidende.*

### SPONTANEOUS RUPTURE OF THE ABDOMINAL WALLS, WITH PROLAPSE OF THE INTESTINES, IN INGUINAL HERNIA.

The patient, a woman 54 years of age, is living in very poor circumstances, her food consisting of potatoes and adulterated coffee. The woman is, therefore, emaciated and anæmic. She acquired an inguinal hernia ten years ago, which has augmented by continuous labor to the form of an oblong sac, hanging down between the limbs. From the lowest point of this sac. a serous fluid began to issue about a year ago. At 7 o'clock in the morning, while visiting a neighbor, the sac ruptured with audible detonation, and she fell down to the floor; but recovering quickly, she took the prolapsed intestines into her apron, and went home. The intestines were exposed to the open air from 7 o'clock in the morning to 2 o'clock in the afternoon, and the patient complained of intense pains in the region of the stomach. There was constant vomiting of greenish fluid. Pulse 86; tongue dry; temperature normal; sensorium free. Two c.m. above the os pubis, and parallel to it, there was a rent in the abdominal walls eight c.m. long. The walls were very attenuated, about the size of cards; the margins of the wound livid, tinged with blood, and very lax. The skin which had formed the sac was full of wrinkles, and as large as a child's head. The prolapsed bowels belonged to the small intestines, the omentum not visible. The loops of the intestines were so numerous, that it could be said with certainty that the whole structure of the small intestines was outside the abdominal cavity. They appeared as a dark blue mass, and they were viscous and cold. The applied apron was saturated with a bloody, serous fluid. The patient rested with her legs on a part of the intestines, and compressed them, but they were not injured. The space into which the intestines had to be removed was diminished by retraction, and it was only by careful reposition of each and every loop that the whole mass could be replaced. The skin was then elevated to a large fold, and deep sutures were applied, with some superficial ones afterwards. While the patient was collapsed and the pulse very low, wine and strong coffee was given. After two days the vomiting had ceased, but there was tympanitis without pain in palpation. The skin was hot; pulse 92; temperature 104; urine passed freely. Four days afterwards the bowels had moved freely, and complete recovery was restored after a short time.—*Scrzst. Intellegenzbl.*

GANGRENE OF THE BLADDER, BY A RETROFLECTED UTERUS, IN THE FOURTH MONTH OF PREGNANCY.

A servant girl, 40 years of age, and not married, suffered from retention of urine for two days. She had always been healthy and the menstruation regular, until two months ago. Since that time she suffered from pains in the lower abdomen. The urine passed but scantily, and she had severe headache when the fæces were retarded. The patient denied energetically having had any intercourse, and the hymen was intact, but there was a round sensitive body in the posterior part of the vagina, and the cervix could hardly be reached. The patient was of a small rachitic form; the face suffering from pains; there was icterus, and the pulse 120; the abdomen very sensitive, and the bladder reached as far as half an inch below the umbilicus. Diagnosis: retention of urine caused by a retroflected gravid uterus. The uterus was replaced by the rectum, and the patient placed on the side forwards. The bladder was emptied every 6 hours by the catheter. On the third day the urine suddenly appeared to be bloody and fetid, and the uterus being constantly found in the fornix vaginæ, a pessary was inserted, but soon withdrawn, on account of pains. Now all the symptoms of peritonitis purulenta set in. After 4 days, a fœtus was expelled with the placenta, but the uterus remained in retroflexion. From this time on, the urine could not be withdrawn by the catheter; the openings in the latter were occluded by dark brown membranes. The spontaneously voided urine had an intensely fetid smell, and gangrene of the bladder was diagnosticated. The autopsy confirmed the diagnosis. There was a good deal of pus among the adherent intestines. The bladder was adherent in its whole length to the abdominal walls, and its fundus reached as far as the umbilicus. It was not possible to separate the bladder from the abominal walls or the intestines. The uterus was retained in the sac of Douglas by fibrinous adhesions.—*Memorabilien.*

FRACTURE OF THE CRANIUM IN A CHILD FOUR MONTHS OLD.

The child fell from the lap of the mother to the floor, a distance of about 75 c. m., and on the left side of the head. Five days afterwards there were symptoms of traumatic meningitis, *i. e.*

convulsions, contraction of the left arm, etc. The child died in a week. There was neither a swelling on the head nor a bloody spot. The autopsy proved the meninges to be filled with pus on the whole surface of the brain, and there was a fracture running from the callous portion of the temporal bone, over the left parietal bone, with a fissure running vertically to the sagittal suture. These cases are very rare, and the physician is often deceived in visiting the child and not noticing any symptoms to the fourth or fifth day, when death quickly sets in.—*Journal de Médecine et Chirurgie pratiques.*

XANTHELASMA.

The patient, a woman 25 years of age, had suffered from icterus for the last two years, and had pruritus. The xanthelasma was found on all parts of the body except the trunk, and it was formed of irregularly marked spots of the size of a bean to that of a half-dollar. They were either at the level of the skin or a little elevated, and of a whitish or yellowish color. On the inner part of the elbow and knee-joints, and on the dorsal side of the hands and feet, there were small nodules, either single or conglomerated in plaques, and only movable with the skin. They were formed by a hyperplasia of the upper layers of the cutis, especially of the papillary body, with enlargement of the papillæ, with local development of fatty masses in the lateral parts of the papillæ, and finally, a development of round, oblong, or otherwise shaped masses of free small granular bodies (pigment).—*Deutsches Archiv. f. Chir. Medicin.*

SOME CASES OF INTOXICATION BY NAPHTHALINE.

A boy 18 years of age, was bitten in the right arm. There was considerable swelling of the arm, but without affection of the glands. A large incision had to be made, and the wound was filled with pulverized naphthaline every day. After three days, there was erysipelas, and the urine contained albumen and formed cylinders. The arm was now put in a warm water bath, and the symptoms disappeared. A hackman 32 years of age, had a large wound on the dorsal part of the foot. The wound was filled every day with pulverized naphthaline. After nine days, the patient suddenly had a high fever; urine dark-colored; headache and loss of appetite. A warm water bath restored the patient. The third case was that of a man whose right foot had been amputated on account of caries three years ago. There was now caries of the toes and the bones of the middle foot on the left side. This foot was then partially amputated, but the flaps from the sole of the foot became gangrenous. After the application of naphthaline for 5 days the wound looked exceedingly well. For about a month he was treated with an ointment containing 25 per cent. naphthaline. Suddenly high fever set in, with considerable thirst, loss of appetite, and prostration; great excitement, with attacks of mania. The naphthaline was not applied afterward, and in a week all the symptoms disappeared.—*Med. Chir. Centralblatt.*

GALL-STONE IN RELATION TO CANCER OF THE GALL-BLADDER.

Carcinoma of the stomach is often found to be developed on the round ulcer of the stomach, and the following case shows clearly that cancer of the gall-bladder is often developed on the lesions produced by calculi, the traumatic origin of cancer being also here corroborated. The patient, a woman 66 years of age, complained of loss of appetite for three weeks, and there were pains in the right epigastric region, the fæces being white and irregular. There was icterus of the skin and conjunctiva, the arteries being enlarged. The liver was large but smooth, its margin rough; in the region of the incisura cystidis felleæ there could be palpated a hard body of the size of a hazel nut, with large margins; the urine contained gall elements. At the apex of the heart a systolic murmur. The gall-stone could be distinctly palpated for three weeks, but after the fourth week it had disappeared. The patient then became œdematous, and on the surface of the liver were now some nodules. The diagnosis was cancer of the gall bladder. The autopsy proved the development to have taken place from the calculus and the scars which were formed by its lesion. There was also cancer of the liver and the right ovary.—*Wiener Med. Wochenschrift.*

A RARE FORM OF RACHITIC THORAX, WITH PERVERSE RESPIRATION.

The child, 13 months old, was in a dying condition when seen, and had the following abnormal formation: Between the line dropped from the sternum and axilla of the right and left side, from the lower margin of the second rib down to the false ribs, the thorax was deeply impressed, forming thus two large cavities. The distance of the lower points of these curvatures was 9 cm. From the middle diameter this arc flattened up and downwards. The form of these cavities was that of a canoe, and it looked as if these lesions had been criminally made by seizing the child with four fingers of each hand on the chest, and the thumb on the back, forcible compression being performed. The brutality of the parents indicated this theory, but by experiments made on animals it was proved to be impossible. The inspiration, which was very short, was followed by symptoms of intense pains, and expiration was sudden and quick. In each inspiration the apex of the thorax became dilated, while the cavities were deepened. The bones of the thorax were so soft, that auscultation and percussion was impossible. The parenchyma of the lungs was nearly entirely destroyed.—*Wiener Med. Wochenschrift.*

ACTINOMYCOSIS IN MAN.

The patient, 21 years of age, suffered from asthma, cough, fever, and emaciation. After a few months several abscesses formed on the back, and there was a pleuritic exudation on the right side. On incision of one of the abscesses, a thick pus escaped, which contained actinomyces. Death followed, by perforation into the bronchial tubes. In the autopsy, the muscles of the right thorax were found to be covered with large abscesses and fistulæ, which were in connection with the right pleural cavity. Right lung gangrenous, and numerous actinomyces were in the abscesses. Left kidney changed into a large actinomycotic mass.—*Zeitschr. f. Allgem. Med.*

ANEURYSMA TRAUMATICUM OF THE RIGHT FEMORAL ARTERY WITH OPERATION IN TWO SESSIONS; RECOVERY.

A farm hand, 23 years of age, was wounded in the right upper leg with a knife. There was considerable hæmorrhage and muscular contractions in the wounded and in the other healthy leg. Compression was made and the leg then bandaged. Repeated hæmorrhages for the next three days, always followed by muscular contractions and convulsions. The patient was then very anæmic, and on the fifth day a pulsation in the wound was noticed.

On the sixth day the contractions and convulsions were most intense, and they were only relieved by large doses of morphine. Compression of the femoral artery below Poupart's ligament. The tumor grew slowly, notwithstanding continued compression and the internal use of ergotine. After a month high fever set in, with hæmorrhage of the lungs. After about six weeks the aneurysm extended from about 6 cm. above the patella to the inguinal fold. There was collapse after seven weeks, and the patient was revived by hypodermic injections of ether. Then it was concluded to make the operation. An incision was made between spina ossis ilei and symphysis pubis in longitudinal direction, and 5 cm. long. The artery was ligatured twice in a distance of 1½ cm. with catgut, no hæmorrhage. The wound was closed by catgut, and antiseptic treatment applied. Four days later, the sac of the aneurysm was opened, Esmarch's bandage having been applied. An incision of 20 cm. was made above the sac and the latter opened. A great quantity of blood clots escaped and there was considerable arterial hæmorrhage, which resulted from the presence of three arterial branches running into the femoral on the posterior wall of the artery and just opposite the wound. After these branches had been ligatured the hæmorrhage ceased entirely. The sac was then filled with carbolized cotton, and three sutures applied. On the next day there was a chilly sensation below the knee, and the pulse varied from 80 to 92 in the next three days The wound on Poupart's ligament healed per primam. Powdered iodoform was applied to the other wound for the next week, but inflammation with gangrene of the patella set in. Boracic acid produced a quick restoration of this part. The patient was in the hospital 197 days, high fever setting in at intervals, but the leg was entirely restored to healthy action, six weeks' treatment with the electric current included.—*Ærzte Intelligenzblatt.*

SYPHILIS OF THE BRAIN.

A telegraph operator, 45 years of age, and of healthy parentage, suffered from chancre and bubo twenty years ago. Six years afterwards there was a protrusion of the tibia with intense pains. Three years later he acquired another chancre, but which healed readily. After two years there was an eruption on the skin of the left side which resulted in a cuneiform ulcer. This eruption spread over the whole body, but no other ulcers were formed. At this time he noticed that his walk became unsteady and he began to stammer. The status præsens is now as follows: The skin of the body is covered with red nodules, aggregated in some parts to circular rings; the left tibia swollen; paresis of the right facialis; the tongue is thrust to the right side; the right palate is a little lower than the left. The bulbus of the right eye is somewhat protracted in its movements; the voice stammering. The right upper extremity shows the symptoms of severe ataxy, so that the patient can bring the hand to the mouth or the forehead by curving it in a certain way. The right lower extremity is paretic. The diagnosis is syphilis of the brain. By the internal use of iod. of potass. the syphilitic psoriasis disappears. After two months the patient is entirely restored.—*Med. Chir. Centralblatt.*

ENCEPHALITIS CONGENITA.

In the medical society of Berlin, Prof. Virchow demonstrated the brains of children with encephalitis congenita, having examined in the last year the brains of 44 new-born children. The neuroglia cells were swollen and partially fatty degenerated. The process is so often found in the inner substance that Petrowski believed the process to be a physiological one. But among the 44 brains only 27 showed symptoms of inflammation, and the pathological character of this encephalitis is evident from the fact that the inflammatory process occurs not only in the diffuse state, but that it is found in groups, the nerve filaments degenerating at these places. A process which produces large degenerative layers is not a physiological one. In France the process is believed to be a nutritive irritation, but this cannot be from the aforesaid facts, and while it is not known that fat can be formed by atrophy of nerves. The process has, therefore, to be regarded as a true ante-partum inflammation.—*Wiener Med. Wochenschrift.*

THE TREATMENT OF URÆMIA.

Since it is known that uræmia is caused by retention of urea in the blood, various and very different methods have been recommended for its treatment. Inhalation of chloroform, purgation by salts, diuretics and injections of pilocarpine are applied to remove the urea in different ways. The diaphoretics are not apt to sufficiently remove the urinary matter by the skin, however large the loss of water may be. Diuretics are without effect in cases of acute nephritis and mechanical obstruction, and it is therefore necessary to consider the anatomical changes in the kidneys in every case. If uræmia occurs suddenly, cold packings and injections of pilocarpine are indicated. If the pulse is small, injections of ether or camphor must be made. The good results of chloroform inhalations in cases of acute uræmia with convulsions are generally known, and they are produced by the excessive exhalation of nitrogen in the narcosis. If uræmia is slowly developed, as in chronic nephritis, the therapeutical means should be of a prophylactic character, seltzers, acetate of sodium and other light salts being given in large doses. If in these cases a sudden attack occurs, the treatment must be regulated according to the pulse. If the latter is suddenly small and thin, digitalis will be the remedy, the cause of the attack resulting from insufficience of the hypertrophic heart.—*Berl. Klin. Wochenschrift.*

THE TREATMENT OF PHTHISIS.

Dr. Edgar Kurz, of Florence, Italy, makes the following interesting statements: The hypothesis of the bacillary origin of tuberculosis and phthisis has nothing to do with its treatment, the anti-bacillary treatment having entirely failed. It is furthermore not proved that a healthy person living in normal circumstances will be infected by a tuberculous person, and in hospitals the phthisical are not in separate wards, as in other infectious diseases. If the bacillus Kochii needs for its development so many different conditions to affect the lungs into which it easily enters, it must be a bacillus quite different from all the others, which develop in a short time and destroy the life. In the miliary tubercles of the peritoneum the bacillus has not been found. Lately arsenic has been recommended in the treatment of phthisis "to break up the bacilli," and one scientist has exactly described the way in which this is done in the body. But arsenic has been given in tuberculosis in Italy for hundreds of years, and its action has been long known, *i. e.*, by improving the breathing and nourishing the nervous and muscular tissue. Milk diet has been recommended for a long time, and it has to be applied in the future, whatever the bacillus may do. Iodoforme has had a good effect in some cases, but the most patients could not stand the smell of the iodoform, and inhalations with it are a torture for patient and physician. In some cases of laryngitis and ulcers of the larynx, the powdered deodorized iodoform may be applied with good results, but only so far as it induces the lungs to gymnastic action. In hæmorrhage the best remedy is ergot in large doses. Against the fever, quinine is the remedy *par excellence*, salicylic acid producing other complications. In some cases where the fever was produced by resorption of pus, inhalation of carbolic acid acted promptly. Kairine was always followed by aggravated symptoms. It is not always commendable to reduce the temperature of the patient, there being certain conditions where a reduced temperature aggravates the other symptoms. The cough is treated with morphine and apomorphine, each 0.05 in 150.0 water. Oil of turpentine, carbolic acid, kreosote and eucalyptus, used as inhalations, are in some cases of beneficial effect. The best inhaler is Oertel's, which works at a distance of two feet from the patient. Forced nutrition by the (stomach sound), œsophageal tubes is very seldom applicable. The night sweat is readily reduced by the old forgotten remedy, agaricus albus, in doses of 0.5 to 1.0. The disease is a social evil, and it will not be reduced by microscopical or chemical examinations.—*Memorabilien.*

### CASE OF INTENSE HYSTERIA CURED BY OÖPHORECTOMY.

Oöphorectomy, *i. e.*, the extirpation of the ovaries, has not produced the results expected, and it has therefore been abandoned by a great many surgeons, but there are some cases where health cannot be restored in any other way. The following case will illustrate this: The patient, a girl 24 years of age, had been healthy to her 11th year, when she had an exanthem of the lower extremities, which returned every winter to her 15th year, and then, with beginning menstruation, disappeared. But intense pains set in with every menstruation, and when 17 years of age, she was taken very sick, so that she had to keep in bed, which she only left after the operation had been performed, *i. e.*, after seven years. There was first vomiting, with pains in the stomach, and all food was rejected immediately. An ulcus ventriculi was then diagnosticated, but after a while these symptoms diminished, and she could take milk and eggs. Soon muscular contraction in the left lower extremity appeared; the upper part of the limb was in extension and abduction while the foot was in plantar flexion. These contractions extended slowly upward; they attacked the left upper extremity and the muscles of the jaw, and the musc. orbic. palpebr. They attacked gradually the other parts of the body, and the latter was then in a continuous state of contraction. Besides that, there was an intense hyperæsthesia of the skin, resulting in clonic convulsions, with loud cries at the least touch. In palpating the ovarian region, the patient closed the eyes, and a gurgling sound was heard in the larynx. The voice was lost entirely, and the patient had to be fed through a small aperture in a defective tooth. By pressure on the ovarian region the symptoms relaxed somewhat, and in a room with dense tobacco smoke the patient felt quite comfortable. This case was similar to that published by Charcot. The patient had to be carried to the hospital in a chloroformed state, on account of the extreme sensibility of the skin. The operation was performed in the usual way, the right ovary being dislocated into the sac of Douglas. Twelve days after the operation, the girl left the bed for the first time for the last seven years, and three weeks afterward she was entirely cured, the symptoms having gradually ceased in the same way as they had come on.—*Memorabilien.*

BLOODY TEARS.

A young hysteric girl complained that she lost a good deal of blood from her eyes every night, and observation proved this to be true. The examination of the eyes showed nothing more than a slight blepharospasm, with facial neuralgia. This is a rare accident, but in the cases of Hasner and Brun the patients discharged a fluid from the conjunctiva which, under the microscope, showed the characteristic blood-corpuscles. The conjunctiva was in all these cases of healthy appearance, and there was neither tumor, nor any other disease of this organ. The accident occurs only in hysterical girls, and mostly at the time of the menstruation.—*Journal de méd. et chir. pratiques.*

NEPHRECTOMY.

The patient, a woman, 21 years of age, suffered from a renal tumor of the size of a man's head. The urine was purulent and there were intense pains. The diagnosis was pyelo-nephritic abscess, originating probably from a calculus in the kidney. Nephrotomy, *i. e.*, a large incision into the parenchyma of the kidney, was first practised, and a large quantity of pus escaped, the patient recovering quickly. But after six months, a fistula having been established, the pains reappeared with the same intensity, and nephrectomy was now decided on. It was tried to separate the cellulo-fatty layer from the kidney, but intimate adhesions prevented this performance. Then the fibrous capsule was incised and the enucleation thus easily performed. The patient had salivation to the amount of a liter every day on the thirteenth and fourteenth days, which was probably produced by a lesion of the pancreas during the operation. After three weeks the patient was discharged as cured.—*Le Praticien.*

A SINGULAR PHENOMENON OF CIRCULATION IN THE CORNEA.

A girl, 17 years of age, complained of pain and a certain tension in the left eye. For the preceding fifteen days the eyesight was entirely lost, but only when the head was kept in a declined position for awhile (sewing, etc.). By friction vision was restored in about two or three minutes. All the organs were found to be in normal condition, there being but slight anæmia and some disturbance in menstruation. The examination of the eye showed normal vision, with but little protrusion. The vessels of the conjunctiva were larger than normally, and on the lower margin of the cornea there was a ring of whitish-yellow color. Lowering the head for some minutes, there was seen a whitish-yellow substance which entered the lower and middle part of the cornea, consisting partially of striated and partially of conglomerated fluid. The experiment could be repeated *ad libitum*, and with the head reclining the fluid could be seen to flow back into the ring. The white veil obstructing thus the eyesight consisted of lymph corpuscles which entered the cornea in a different manner. The girl was treated with iron, etc., and sent to the country, where the menses becoming normal, the phenomenon disappeared entirely.—*Annali di Othalmologia.*

CHRONIC TUBERCULOSIS OF THE CHOROIDEA.

A young boy of 17 suffered first from a swelling of the right knee and of the carpo-metatarsal joint of the left thumb. Three months later, vision of the right eye became imperfect and he had fainting spells. After one of these the right eye was found to be entirely blind. Two months later a tumor developed in the inner part of the right eye, and it reached the size of 16 mm. vertical and 18 mm. horizontal diameter, extending to the cornea. It was elastic and painful, and had a rough surface, no connection existed with the anterior chamber. Later the tumor opened, and the eye was then enucleated. Cornea, iris, and lens healthy, retina well preserved. The tumor is in the choroidea, and of a conglobate tuberculous character, with round and giant cells. Inoculation of rabbits proved the true tuberculous character of the tumor, but there were no bacilli found.—*Correspondenz-Blatt f. Schweizer Ærzte.*

GLYCERINE IN FEVERS.

Glycerine is in a certain sense a nutriment, and while it can be given in large doses with small parts of beef-tea and milk it will give the patients the power to resist the attacks of the fever. The best method to give it is with citric acid, the taste being then a very agreeable one. The examination of the urine after the use of glycerine shows a considerable decrease of urea. If the glycerine is not given for a certain time, the amount of urea increases again. 30 parts to 150 water can be given, tablespoonful every hour.—*Revista clinica e therapeutica.*

### A CASE OF IDIOPATHIC MILIARY TUBERCULOSIS OF THE SPLEEN.

It is generally accepted that miliary tuberculosis of the spleen is a secondary disease originating from the tuberculosis of other organs, and especially from the lungs. The following case will contradict this theory: A prisoner, 25 years of age, had suddenly an attack of fever, with rising and falling temperature, intense headache, and cough with expectoration of mucus. In auscultation murmurs were heard all over the lower part of both lungs; the diameter and position of the heart normal, also its action. The abdomen somewhat tympanitic, liver normal, spleen greatly enlarged. The headache and cough increased, the face became cyanotic, there was hæmorrhage from the nose, diarrhœa, and retention of urine. After a few days delirium set in, and the patient tried to escape. The patient died after 17 days. The autopsy showed the lungs to be in a perfectly healthy condition, as were the heart, the stomach, and the intestines. The spleen, about three times larger than its regular size, contained in the capsule and the interior part numerous miliary tubercles adjoining the vessels.—*Aerztl. Intel. Blatt.*

### POISONING BY CHLORIDE OF POTASSIUM.

It is not generally known, even among physicians, that chloride of potassium in large doses has strong poisonous properties. Dr. Friedlænder has made ten autopsies in the last three years where death was caused by the use of the chloride. The following two cases are lately reported: A child, four years of age, on the recovery from scarlatina and dropsy, was given a solution of two drachms of the chloride in three ounces of water and one ounce of syrup, every two hours a teaspoonful. After taking about half of the mixture vomiting with diarrhœa set in suddenly, and cyanosis and death followed shortly afterwards. The autopsy showed all the symptoms of poisoning by the chloride in the very emaciated child. A strong man, 49 years of age, was advised to dissolve a teaspoonful of the chloride in a glass of water, and to take of this solution a tablespoonful every two hours. By a misunderstanding upon his part, he took every two hours the whole mixture, containing one teaspoonful of the salt, and he swallowed thus 50 grams in 36 hours. He died on the third day, and the autopsy proved the cause to be poisoning by the chloride of potassium. (Two distinct cases of poisoning by the chloride occurred in my practice 9 years ago, the druggist taking in each case the same amount of grammes as were prescribed for in grains. Tr.)—*Med. Chir. Centralblatt.*

### INFLUENCE OF DIPHTHERIA ON PREGNANCY.

Although numerous works treat fully the subject of diphtheria, no allusion is made to its influence on the products of conception. Dr. A. Ollivier has had occasion to observe in the Hôpital Saint Louis a case of abortion in a woman four months pregnant, following a diphtheritic angina. The diagnosis and progress of the case was: Diphtheritic angina; extension of the pseudo-membranes to the nasal fossæ, to larynx, to trachea, and to the bronchial tubes; abortion; paralysis of the soft palate; imminent asphyxia; death. The disease was grave, not only in its nature, but also in the fact of pregnancy and the puerperal state following the abortion. How was, in this case, the abortion produced? What was the mechanism? At first we can explain it by the intensity of the fever. The influence of the febrile state on the course of pregnancy is not to be doubted; it has been demonstrated clinically and experimentally by Kaminsky. According to him, the high and prolonged elevation of temperature is one of the frequent causes of death of the product of conception in the infectious diseases. A temperature of 42°, 42.5° C. in the human being, of 41.5° C. in the rabbit, is absolutely deadly to the fœtus, and in women the danger of death of the infant commences at 40° C., and increases proportionately with the temperature. But our patient never had an intense fever; for, on the same day as the abortion the thermometer only marked 38.04° C. Therefore the temperature was not the cause of the abortion. On the contrary, the author finds the cause in the state of the maternal blood, either insufficiently oxygenized and saturated with carbonic acid, or altered by an infectious germ. In other words, the abortion was due to asphyxia in the first hypothesis, to a true intoxication in the second. The product of conception, once dead, is nothing but a foreign body for the womb, and therefore expelled. Now, which one of the two causes had more influence on the abortion? This occurred in the night of the sixth or seventh day after the commencement of the disease. On the fourth day the respiration was easy; on the fifth became labored; on the sixth the cyanosis appeared at the lips and at the extremities. Therefore it must be admitted that the asphyxia played a certain role; this role was not predominant, and the author believes that in this case we must recognize the cause in the diphtheritic poisoning; that this poisoning was primitive or consecutive to the local infection.

The nature of the infectious and specific agent of diphtheria is yet unknown. Is this agent parasitic or not? The partisans of the former opinion are the most numerous. It is known that in diphtheria the blood undergoes notable changes. Its respiratory capacity considerably diminishes. And it has been shown by Hueter and Tommasi, by Letzerich, by Oertel, etc., that the diphtheritic false membranes, the lymph, the blood, and the kidneys themselves, abound with vegetable organisms, by some considered as the infectious agent, and by others simply as accidental elements. Uutil lately, the negative experiments of Brauell, of Davaine, of Bollinger, on the blood of embryos of different animal beings, seemed to have demonstrated that in the splenic fever the bacteria could not pass from the mother to the embryo. But at present this opinion is recognized to be false. Strauss and Chamberlain have experimentally proven that the bacteria of splenic fever can pass through the villi of the placenta and infect the fœtus. Cannot the same happen in diphtheria? The blood of the fœtus may become infected by the bacteria of the maternal blood. Concluding: diphtheria can in pregnant women be the cause of abortion, and acquire by it a greater gravity. The abortion will be due, in a great number of these cases, neither to asphyxia nor to the elevation of the temperature of the blood, but to an alteration of this fluid, which is incontestable. The possibility of an abortion, with its dangers, command us in the future to take all measures of precaution and of isolation whenever pregnant women are living in the same halls containing patients affected with diphtheria. It is particularly on this point that Ollivier desires to call the attention of his colleagues.—*Archives Générales de Médecine.*

HOANG-NAN AND ITS THERAPEUTICAL INDICATIONS.

In 1879, M. Lesserteur, a missionary in Tonquin, and Director of the Seminary of Foreign Missions, published an account of hoàng-nan, a Tonquinian remedy for hydrophobia, for lepra, and other affections, and with the aim of making known this new remedy, he experimented. In 1881, Dr. Barthèlemy published simultaneously in the *Journal de Médic. de l'Ouest* and in the *Bull. Gén. de Thérap.*, a complete study of hoàng-nan, and the results obtained in the treatment of several affections of the nervous system, and of a case of varicose ulcer declared incurable. In 1883, the same author published in the *Bull. Gén. de Thérap.* a new study, eulogizing the hoàng-nan, especially as a specific for hydrophobia, giving at the same time the history of a case. In his paper he divided into three groups the diseases in which the Tonquin bark ought to be again experimented with: Diseases of the nervous system; poisoning by virus and venoms; local and constitutional cutaneous affections. The action of the remedy in the last group is probably due to its activity on the cerebro-spinal system. Brucine and strychnine, which enter into the constitution of this bark, are well-known stimulants of the nervous system, and, on the contrary, all the grave accidents due to an absorption of a virus or venom, depress profoundly this apparatus. Not long ago, Pasteur communicated to the Academy of Medicine that the cerebro-spinal axis is the principal seat of elaboration of the rabid virus. Could it not be the same with the venom of serpents? In four cases observed by Barthèlemy, Viaud-Grand-Marais and Albert Malherbe, the hoàng-nan was employed in the prodromic period, and the result was most brilliant; no rabid accident whatever was observed. The want of success is mainly due to the late use of the remedy *in extremis*, and to the insufficient doses. Barthèlemy recommends the hoàng-nan to be given in increasing doses, and especially during the period of incubation, and large doses in the confirmed cases of hydrophobia. One of the first results is to quieten the *moralè* of the patient and to gain his confidence. Another consequence of the employment of the remedy is to permit us to diagnosticate to a certain point if we have to deal with a confirmed case of rabies. The cures obtained of patients bitten by the cobra di capello and by the black viper, are one of the reasons that brought us to think that, equally with the virus of hydrophobia, the venom of serpents might have the cerebro-spinal axis as the principal seat of elaboration. Another group of diseases amenable to treatment by hoàng-nan, is that of certain local or constitutional cutaneous affections, syphilis excepted; it seems that the Tonquinian bark has no effect on this virus. It was used in a case of eczema of the scrotum, invading the perinæum and the superior and external part of the thigh, and of ten years' standing; it was cured in a few days. Are not these cases, and many others analogous reported by exotic experimenters, sufficient to excite us to thoroughly study the hoàng-nan and its therapeutic properties?

CYSTICERCUS IN THE VITREOUS HUMOR.

Mlle. X., aged twenty-three years, consulted Prof. Dor on the 24th of September. Before last June, she possessed a good sight. On the 2d of June, she noticed a little cloudiness before the right eye, but not enough to prevent her seeing the minutest objects. From the 10th to the 12th of the same month the sight of the right eye seemed to diminish, and she commenced to see *muscæ volitantes*. But her ailment was attributed to nervous troubles. In the month of July, she saw a round, black macula, and by closing the eyelids she could perceive a constant, fixed, and brilliant point. In the month of August, the black macula had become translucent from the center to the periphery. Finally, the sight becoming more and more feeble, she consulted Prof. Dor. He found a detachment of the retina, and a round, whitish, translucent body adherent to the infero-internal part of the retina, having the aspect of a displaced cataract, which he recognized as a cysticercus. Having chloroformed the patient, Prof. Dor made a vertical incision, 8 millm. long, and was enabled after four or five attempts, to withdraw the cysticercus. It is to be noted that after the incision, and during the operation, no vitreous humor escaped. The hæmmorrage was insignificant. Atropine was applied daily. On the 12th, the patient was discharged cured, although with an almost complete detachment of the retina. The entozoa was found to be the cysticercus cellulos.æ These cases are rare. This is the only case that the Professor has seen in 14,000 patients treated by him. Prof. Gayet has found one case of subconjunctival cysticercus in 9,000 patients. Profs. Hasse and Eberth, of Zurich, have never found it. It is very rare in Switzerland, and relatively frequent in Germany. —*Lyon Médical.*

### SUGAR AS AN ANTISEPTIC.

Dr. T. Fischer announces in the *Centralbatt für Chirurgie* that since last May he has used in the surgical clinic of Strasburg, cane sugar as an antiseptic. It can be used, mixed with naphthaline, equal parts, or with iodoform in the proportion of 1 to 5. The mixture is placed on the dressing over the wounds properly closed with sutures ; where the epidermis is wanting the sugar is applied directly. During the operation the wound is to be disinfected by a solution of sublimate, 1 to 1000. The treatment with the sugar can be left in place, 8 to 15 days without the sugar be dissolved. The wounds treated with sugar presented a good aspect, the bandages did not hurt, and no bacteria could be detected. The granulations rapidly developed, no propensity to hæmorrhage, and cicatrization formed with rapidity. In the sutured wounds healing always occurred *per primam intentionem.* The author has studied the following questions: Is the sugar decomposed ? What are the formed products ? Dr. Fischer believes, by his results, that the sugar dressing is to be recommended, and he advises its use as to be able to fix its proper value.

### ALTERATION OF THE INFERIOR VENA CAVA IN HEPATIC CIRRHOSIS.

Frequently it is noticed in some patients suffering with hepatic cirrhosis that the œdema of the inferior limbs precedes the development of the ascites instead of being consecutive to it, as is taught by classical works on the practice of medicine. Professor Giovanni finds the explanation of this anomaly in the alterations of the vena cava, which frequently, according to him, are the result of cirrhosis of the liver. Numerous autopsies have enabled him to recognize that this disease is complicated very frequently by hyperæmia of the walls of the inferior vena cava, by phlebitis and varicose dilatations, all which lesions have never so far been demonstrated before Dr. Giovanni, and naturally sufficient to produce disorders in the circulation of the inferior limbs to explain the œdema.—*Gazzetta degli Ospidali di Milano*, 1883.

TREATMENT OF POLYPI OF THE EAR WITH ALCOHOL.

Dr. Politzer states that he has obtained good results by dropping alcohol several times a day into ears affected with polypi. The alcohol ought to be applied at least three or four times a day without interruption. After continuing it fifteen days to two months the polypus commences to contract, and gradually diminishes in size to a complete disappearance. The treatment was equally successful in fibrous polypus. During the first days the patients felt an excessive pain, and later an increase of heat in the affected part.—*Wiener Med. Blatt.*—*Revue Médicale.*

BERIBERI.

Dr. Scheube has described the four following symptomatological groups relating to beriberi: First, sensitive or motor paralysis, frequently in the paraplegic form, with muscular atrophy; second, affections of the heart with predominance of asthenia; third, dropsical affections; fourth, diminution of renal activity. The author considers this disease as a specific neuritis following a subacute course. Dr. Wervic, on the contrary, considers it as caused by an alteration of the blood, a sort of anæmia.—*Deut. Arc. Klin. Med.*—*Revue Médicale.*

THERE is living in Montijo a woman with four mammary glands; two are situated in their ordinary place, and the other two, a little smaller, perpendicularly and two centm. above, one on each side, with their corresponding nipple. She is at present nursing a child with the four breasts, all having an abundance of milk.—*Il Siglo.*

### The Treatment of Chronic Urethritis and Blenorrhagic Cystitis.

If the patient is scrofulous or tuberculous he ought to be treated accordingly with arsenic, iodine, or cod liver oil, and at the same time small doses of the bals. copaiv. have to be administered. The local treatment consists of injections, and instillations, cauterization or catheterism. Injections are often useless in chronic urethritis, the seat of thə inflammation being in the cul-de-sac of the urethra, where the injections cannot enter. The new syringe of Guyon, where the injected fluid flows back by a second apertureor tube, is doubtless accompanied by good results when the inflammation is in the anterior part of the urethra. Instillation is followed by very fair results in chronic urethritis. The instrument used for this purpose is an explorator of hard rubber, with a fine aperture at its point. To this is attached akind of Pravaz syringe tightly fitting and exactly graded. If an instillation into the posterior part has to be made, the sound is introduced into the latter, and after the first resistance has been overcome (the bulbar part) it is thrown into the cul-de-sac, where there is complete resistance. It is then thrown back a little, and twenty drops of a strong solution of the nitr. of silver injected. The instrument is kept *in situ* for three or four minutes. The treatment has to be continued for fifteen to twenty days. The application of bougies or catheters is often followed by damaging results.—*Journal de Méd. et Chirurg. Pratiques.*

### Miscellaneous.

In some parts of France the following curious customs are in vogue up to to-day: When a woman is parturient her corset is hung on the bell of the church and the latter rung three times. If the after-birth does not come, the woman's head is covered with the husband's cap turned inside out, and then she must blow in a big bottle. The father of the new-born child must report the birth in the court-house. When a boy is born, he wears a stiff hat, but if it is a girl he appears in a lady's bonnet.

CREMATION is officially advocated in Portugal. In cases where there is a will made that a person is to be buried in the ground, the body is exhumed after five years and then cremated.

A DOCTOR, with an Arabo-Hindostanian name settled in one of the suburbs of Paris. In a short time he had a large run of wealthy patients. The police sent an officer to inquire about the diploma of the mysterious practitioner. The doctor received the officer very politely, and smilingly showed him his full certificates and diploma from the University of Paris. "But," he said to the officer, "you will oblige me if you do not speak about this affair, for I would lose all my patients in a short time, if they should know that I am a regular Parisian physician."

THE members of the school of Catholic medicine (!) at Lille, France, have opened a subscription for the erection of a chapel in the Basilika of Montmartre, where they will pray "for the souls of those *confrères* who went astray from the right path of Catholic medicine and religion, and who, being atheists and politicians, are damned forever to infernal depths." As the chapel will cost 50,000 francs, and as there is but little money at present, it is doubtful if the "political confrères" will have the chance to be prayed for.

A CASE of rather extravagant character came before the Superior Court of Bordeaux, France. A regular physician, but one who had largely advertised a "sure cure" for sterility, was engaged by a lady, and he performed his operation (artificial impregnation). There was no result, and the lady refused to pay the price of 1,500 francs. The doctor brought suit against the lady and her husband, but the court decided that the operation was not a regular one, and the doctor had to pay the costs.

A CASE of congenital ectopia of the heart came before the Paris Academy of Medicine. The woman had a bifid sternum, and the diaphragma was divided in its anterior part, so that the heart could be felt pulsating through the thin walls of the chestcovering, which was pigmented. The ventricles are small, and the axis vertically directed, so that the whole hangs down to the abdomen. There was also a small umbilical hernia.

SALICYLATE of zinc is, according to the *Independencia Medica* of Barcelona, an excellent remedy in cases of ulcerative cancers, the properties of antiseptic and astringent action being combined in this remedy.

### DO THE CONTRACTIONS OF THE UTERUS DEPEND UPON THE CEREBRO-SPINAL SYSTEM?

It has been known for a long time that women with total paralysis of the bladder and rectum, had normal labor-pains and regular deliveries. Dembo, in St. Petersburg, has made experiments on the connection of the cerebro-spinal and uterine nervous systems, and he aims to prove (1) that there are points in or around the uterus which, when irritated by the electric current, provoke normal contractions of this organ; (2) that these contractions will also be produced after the uterus has been disconnected with the cerebro-spinal system, the animals, mostly rabbits, having been either curarized or chloroformed. The author, after giving details of his experiments, says further: (1) There are regions in the anterior vaginal wall which, by the electric current, will provoke contractions of the uterus; (2) the same contractions are produced by the galvanic currents; (3) these contractions can be provoked several hours after the death of the animal; (4) the uterus of young animals is more liable to contract than that of older ones; (5) the two nervous systems of the uterus of rabbits are independent from each other; (6) in pregnant animals the irritability is diminished; (7) the uterus of very young rabbits is also liable to be contracted; (8) curare diminishes the contractibility. Dembo thinks, therefore, that the uterus is entirely independent of the cerebro-spinal system, and that it has special nerve-centers. (The uterus of the rabbit cannot be compared with the uterus of the human female, the former being the most important organ of this animal).—*Comptes Rendus de la Société de Biologie.*

### ECHINOCOCCUS ALVEOLARIS OF THE LIVER.

The patient, 29 years of age, has had icterus four or five times, once dropsy, and five years ago intermittent fever. At the present time the skin is intensely icteric; lungs and heart healthy; liver greatly enlarged and sensitive, its surface full of nodules; the spleen also enlarged; the abdomen is tympanitic; the urine shows a large amount of gall-products. The patient complained of pains in the abdomen, as if a stone were laying there; and there was also xanthopsy. The diagnosis was cirrhosis of the liver with icturus. The patient died after five days. The right lobe of the liver was enlarged, and of a greenish-brown color; on its inner surface there were small nodules of a hard consistence, which contain alveolared echinococci. The left lobe was adherent to the diaphram by connective tissue filaments, its capsule enlarged and rough on its surface. The tumor on the liver was of the size of a child's head, and of light color. It was as hard as bone, and its stroma filled with alveoles of the size of a pea, with irregular borders. The microscopical examination of the tumor showed that there was not a vestige of liver tissue proper, but there were numerous minute alveoles, which were inhabited by echinococcus sacs. The lamelar layer, consisting of chitine, was not adherent to the walls of the alveoles. In water, it was observed that one sac was often formed by two or more alveotes, which could be separated. On the other hand, there were often communications between two or more alveoles. On the margin of the tumor the number of alveoles decreased, and there were large filaments of connective tissue which looked like the acini of the liver. The contents of these filaments were often colored by a yellowish fluid, which was similar to that of the gall-bladder.—*Aerztl. Intelligenz-Blatt.*

### ON COMBINED ŒSOPHAGOTOMY.

For impermeable strictures of the œsophagus, gastrotomy has been hitherto the only relief. Gussenbauer has lately performed the above-named operation on two persons, and in both cases he restored the function of the œsophagus. The first case was a stricture caused by swallowing sulphuric acid, and it extended from the level of the ring cartilage to the upper chest part of the œsophagus, at about the bifurcation of the trachea.

He made first external œsophagotomy (Guattani). While the whole mucous membrane was deformed by scars, and dilatation had been practised without success, he introduced a small pointed sound of 1 mm. diameter through the stricture, over which was pushed another hollow sound. Withdrawing the pointed sound, he cut with a herniotome to the right and left, no hæmorrhage following. Instantly he could introduce a No. 8 catheter, by which a fluid was injected into the stomach. The patient was nourished by a stomach sound of the largest caliber. The wound of the throat healed quickly. The second case was that of a child two weeks old who had been given carbolic acid. The external incision was made a little lower than usual, and a sound of $\frac{1}{3}$ mm. diameter was introduced; afterward the hollow sound, and the operation made as above. A No. 12 catheter could be immediately applied. The wound healed in 35 days. The child, nearly starved to death before the operation, is now in a flourishing condition, and the father inserts a bougie but once a week. —*St. Petersburg Med. Wochenschrift.*

REFLEX DILATATION OF THE PUPILS A SYMPTOM OF SOME AFFECTIONS OF THE BRAIN AND MENINGES. Translated by P. E. ARCHIBALD, M.D., *Gazette des Hôpitaux.*

Professor Parrot shortly before his death made the following discovery, referred to by Dr. Landouzy in his lectures on general tuberculosis. In young children affected with certain diseases of the brain and its coverings, giving rise to coma with or without convulsions, and accompanied by pupillary contraction, stimulation, as by pinching, of the skin of the epigastrium during the stage of coma, produces a more or less marked dilatation of the pupils. In other cases, also characterized by coma but without convulsions, no amount of cutaneous excitation can induce this phenomenon.

The diseases in which this sign is present are marked by normal or increased cutaneous excitability; they are the following, demonstrated by a number of examinations made on the cadaver: tubercular meningitis, hemorrhages underneath the pia-mater, some cases of chronic hydrocephalus and a few cases in which no appreciable anatomical lesions were found. All this group is characterized by compression of the cranial contents.

The cases ln which this dilatation is absent present cutaneous anæsthesia and a non-compression of the encephalon, as evidenced by depression of the fontanelles. The most important of these are: œdema of the pia-mater, congestion of the nervous mass, and of the meninges.

Now, physiologists assign two sets of causes for the motion of the iris. The first purely nervous: contraction of the pupils due to the circular fibres of the irides supplied by the 3d pair of nerves, dilatation caused by the radiating fibres of these muscles controlled by the sympathetic. The second cause is vascular; an increase of blood to the irides giving rise to contraction and a diminution to dilatation of the pupils. Professor Parrot thought the phenomenon observed by him must be due to vascular action, and explained it in this way: the stimulus produced by pinching the skin is transmitted by the afferent nerves to the centres in the medulla and thence reflected to the vaso-constrictor of the irides, thus causing a diminution in the calibre of the vessels of these mnscles, a diminished supply of blood, and hence pupillary dilatations. The only practical deductions from the above so far are: that children affected with coma with or without convulsions, in which the pupils do not dilate on repeated cutaneous stimulation, do not suffer from tubercular meningitis nor from hemorrhage of the pia-mater.—*New Orleans Medical and Surgical Journal.*

FOREIGN BODY IN THE UTERUS.

Dr. E. Hjertstrom published in the *Hygiea* a curious case of a woman, aged 49, subject since puberty to periodical attacks of mania, with lucid intervals. She was admitted to hospital on account of an abundant and fœtid vaginal discharge. Examination of the uterus showed the neck covered with granulations, and irritated by a secretion coming from the uterine cavity. The exploration of the cavity revealed within it a metallic body. Extraction being indicated, after the dilation of the cervix, the doctor removed a piece of metal lodged in the superior part of the uterine cavity. This metallic body proved to be the brass socket of a candlestick, measuring 0 m .015 in length and 2 centim. in diameter and 4 centim. of diameter at the rim. The patient could not state how the socket entered her womb; menstruation had ceased five years ago, and the last labor dated back twelve years. She did not remember having any symptoms of parametritis; she had had some uterine pains. At what time did this body enter the uterus? After the last parturition, before the womb had contracted! That is not very probable. We rather think that the piece detached itself from a candlestick introduced in the vagina, and that it was seized and retained while the uterus was contracting during its mechanical excitations.

### PROPERTIES OF ADONIS VERNALIS.

This plant has been for a long time, in the south of Russia, a popular remedy for dropsy, but has not been yet submitted to scientific researches in order to determine its physiological and therapeutical action. Professor Botkin has largely experimented with it in his clinic at St. Petersburg, and Dr. Bubnoff has published a report of the results obtained (*Allgemeine Med. Central Zeitung*). This remedy has no effect in any dropsies except those caused by a cardiac affection. After the administration of the adonis vernalis the beats of the heart become more distinct, the volume of the organ diminishes, and the sounds are more audible. The systolic souffle of the aortic insufficiency especially becomes more intense. The rhythm of the heart is more regular, the pulse slower and more forcible. The urine excreted in the 24 hours notably increases from 300 or 350 grams to 550 or 600. In case there is no alteration of the kidneys, the albumen and the epithelial tubules disappear. The œdema disappears in proportion as the urine increases. The remedy is given in infusion in the dose of 4 grams in 180 grams of water, flavored by two drops of oil of peppermint. A tablespoonful of this infusion may be given every two hours.—*La France Médicale.*

### SPINAL MENINGOCELE WITH SPONTANEOUS RUPTURE AND RECOVERY.

A child ten years old has a tumor of the size of an apple on the lumbo-sacral region, the origin being dated back to its birth. The tumor has been tapped by several physicians, but it always rapidly refilled. The actual state is as follows: The tumor is soft, the covering skin not being colored and it is provided with a small pedicle. Its volume is changed by a rapid diminution in case the patient lays on its abdomen, and is increased again in the vertical position and in defecation. It is transparent as a hydrocele. In touching it, no heat can be perceived and palpation causes no pain. It is fluctuating and reducible and by taxis a part of the fluid re-enters the spinal canal. The latter procedure is accompanied by cerebral phenomena, the child complaining of dizziness in the head and somnolence. On the bases of the tumor, or rather at the level of its pedicle there is a depression in the form of a fold by which the fluid escapes. It is, of course, congenital and a meningocele containing only cephalo-rachidian fluid. These tumors are very dangerous on account of causing instant death. A fine trochar was introduced; 250 grm. of a clear fluid withdrawn, and a tight corset applied. A week after, the same puncture was renewed, and this operation several times repeated, but the tumor grew apparently larger than before, and attained the size of a child's head. There was now mortification of the skin and a scab formed which fell off and the tumor burst. The child was laid on its abdomen for 28 days, a large amount of fluid escaping every day. After three months there was but a small fistula, and after six months the fistula was closed by thickened epithelium. The aspect of the former tumor is that of a patch of thickened skin without any opening, and the child is entirely healthy.—*Archives Générales de Médecine.*

CEREBRAL SYPHILIS.

Is either characterized as gummous phagedena or an arteritis. The first is caused by a nocturnal headache followed by partial convulsions and coma or cerebral paralysis, especially of the third pair, an incomplete hemiplegia and paretic troubles. The syphilitic arteritis is mostly limited to the sylvian arteries at their bifurcation and to the basilar trunk. The vessels are slightly enlarged, which is due to yellowish nodules found in the walls of the artery. It is mostly accompanied by vertigo, cephalalgia, end pupillary dilatation.—*France Médicale.*

LARYNGITIS HYPOGLOTTICA HYPERTROPHIA, TREATED WITH TUBES.

Schroetter first recommended the use of tubes in cases of certain affections of the larynx, but the reported results seemed not to have been satisfactory. The following case will show the importance of the application of tubes in certain conditions. The patient, a women 35 years of age, had always been healthy, menstruated at 16, and married at 20 years of age. In fourteen years of married life she had seven children. Eight years ago she noticed a hoarseness, which temporarily disappeared to reappear again. In the last year the symptoms became grave, breathing being difficult, and the voice disappearing entirely. At last asphyxtic symptoms made it necessary to bring the patient into the hospital. The face is cyanotic; stertorous breathing audible at a long distance; voice soundless; larynx and trachea powerless in palpation, and neither enlargement nor dislocation visible; lungs healthy, as is also the stomach. The laryngoscopic examination shows anæmia of the soft palate, of the epiglottis, and of the entrance of the larynx. In phonation the slightly red-colored vocal cords do not join each other, but they seem to be agglutinated to a swelling, which fills up the internal larynx so that there is but a small opening on the angle of the glottis, and not larger than a pencil. The whole swelling appeared white without vascularization. The free margin is uneven and especially on the left side, it seems to be ulcerated. In inspiration the vocal chords move and the swelling is seen to be subchordal. The diagnosis is laryngitis hypoglottica hypertrophia, the absence of any pain excluding cancer. There was no symptom of tuberculosis. Catheter No. 10 was introduced the first day, and kept in situ for four minutes. In the following night the patient complained of dyspnœa. Four days afterwards catheter No. 11 was easily introduced and kept in situ for five minutes. The next week catheter No. 12 was applied. Now the laryngoscopic examination showed a different picture. The swelling adjoining the laryngeal-mucous membrane had disappeared, and on the left side there were seen granulations. Later, Schrœtter tube No. 1 was introduced, and then after a short time tube No. 3 and 5 constantly applied. In the meantime the woman had learned to apply the tubes herself, and she did it with such exactness that the complete cure was left to herself. Two months after the beginning of the treatment inspiration and aspiration are normal and the voice hoarse only a few hours after introduction of the tube. The swelling is nearly gone and the vocal cords move freely.—*St. Petersburg Medical Wochenscript.*

THE PATHOLOGY OF OPHTHALMIA JEQUIRITICA. Translated by C. R. EGGEMANN, M.D., Detroit, Mich.

Prof. Sattler in *Zehender's Klinische Monatsblaetter fuer Augenheilkunde* says: Between the application and the first appearance of subjective or objective symptoms, there is a period of incubation of about three hours, increasing slowly at first then very rapidly so as to produce a violent ophthalmia within sixteen hours. The lids are agglutinated, œdematous, so as to be even or project above the orbital edge, glistening, hot, and very painful on touching. The palpebral conjunctiva is covered with a thick, tenacious, grayish-yellow membrane. The retrotarsal folds and the ocular conjunctiva are œdematous, the former dark-red in color covered with a fine grayish-yellow membrane, the latter red, slightly tinged with yellow, and surrounding the cornea like a wall. The patients are uneasy, complain considerably, and the temperature is elevated. Occasionally there is swelling of the præ-auricular gland accompanied with a severe coryza. Twelve to sixteen hours after an application of one per cent. infusion the inflammatory process reaches its height, and continues for a day. The membranous exudation could be removed with ease from the palpebræ. The secretion is profuse and purulent. On the fifth or sixth day, the formation of this membranous exudation ceases, only at the retro-tarsal folds it adheres for a few days longer, leaving in some cases a slight cicatrix. It takes weeks before the conjunctiva has regained its normal appearance, and the last symptoms of redness, unevenness, and the dirty yellowish discoloration have disappeared. In some cases there were superficial opacities and shedding of the corneal epithelium. These are the symptoms of ophthalmia jequiritica produced by a single application of an active infusion on the normal conjunctiva of man. By an active infusion is meant the maceration of the pulverized seed, after the shells have been removed, for twenty-four hours at an ordinary temperature, having the strength of one or five per cent., and to be used immediately after filtering. We can limit the intensity and duration of the ophthalmia by the number of applications made. Age diminishes the action of an infusion and in time it becomes inert. Maceration at high temperature destroys its action, while maceration in ice-water for twenty-four hours did not affect it at all. A few remarks on the experiments on rabbits: the lids were œdematous, felt hard as a board and in some cases the sloughing was partial or complete. The membranous exudations on the palpebral conjunctiva are denser and tougher and on the retro-tarsal folds have a diphtheritic appearance. After the disappearance of these symptoms we have symblepharon or entropion remaining. The cornea is always implicated if the ophthalmia attains any degree of severity. Diffuse opacities may remain, or the cornea may become completely necrosed. A number of the animals succumbed after the inflammation extended from the lower to the sternal region, which, on section, appeared to be a waxy infiltration. An animal that had undergone this process, and with considerable change in the conjunctival tissue, could not be inoculated again so readily, strong infusions exciting only a slight conjunctivitis. Albumen and coloring matter, sometimes other things, are taken for micrococcus; then Moura Brazil speaks of conidia (should be gonidia), mycelium, fibers, and spores, of which there is not the slightest trace to be found in the infusion. The same gross error is shown in the examination of the detached membranes. Prof. Sattler found a quantity of albuminous matter differing materially from that of the other leguminosa. Prof. Hilger obtained a crystalline substance from the seed soluble in slightly alkaline water, applied to the normal conjunctiva in the strength of one per cent., it was inert. A ferment was not found in the infusion. The microscopical examination by Prof. Sattler shows that a bacillus in enormous quantities is found in the infusion immediately or a few hours following filtration. The bacilli have a cylindrical, homogeneous, opaque form, and an oscillatory or rotary motion. The infusion is of a greenish-yellow color, as a rule had a turbid appearance after being filtered, a peculiar, characteristic odor, becoming disagreeable in time. After standing a few weeks the infusion slowly clears up, and has an odor resembling fresh lime. If we test the infusion for albumen at this time, we find that it has completely disappeared.

The temperature has considerable influence on the formation of the bacilli. High degrees destroy them entirely, low degrees check them, but they continue to form on being brought into the ordinary temperature of a room ; 34 to 36 degrees C. is the most favorable, bacilli beginning to appear after a maceration of twelve hours. On filtering the infusion and drying the sediment, the bacilli remain latent, but on moistening they become active. A submersion in a weak corrosive sublimate solution (O. I. P. M.) not to exceed two or three minutes does not destroy them. In this dry state they have withstood the temperature of 110 degrees C. for five minutes. The moistening and boiling for five minutes suffices to destroy their activity. In power of resistance these bacilli are far behind the bacilli obtained from an infusion of hay. In the discharge as well as in the membranous exudation the bacilli are met with, but not in large quantities. The secretion will produce an ophthalmia if brought into a normal eye, but the inflammatory symptoms are not so intense. The bacilli are more numerous in the infiltrated conjunctiva and in the fibrinous exudation of the subconjunctival tissue of the retrotarsal folds during the first stage of inflammation. To render the infusion innocuous, it was necessary to boil it for half an hour. After filtering and exposing the infusion to the air, the bacilli were poorly developed and ineffective. A solution of corrosive sublimate, 1 to 8,000, destroyed the germs. The reason for using it in this strength was on account of the albumen the infusion contained. Thymol, 1 to 2,000, was ineffective, 1 to 1,100 sufficed to render them innocuous. Iodoform added to the infusion during maceration, and after filtering, did not check the development of the bacilli, nor of the ophthalmia. The dusting of iodoform into the eye did not retard the progress of the ophthalmia. A diminution in the intensity of the ophthalmia was accomplished. The propagation of this bacillus upon serum, gelatine, etc., was successful, retaining its peculiar physiological action when brought into contact with a normal conjunctiva. The hypothesis which remains for us to accept, is, that there is a widely diffused and benign bacillus, the spores of which gaining access to an infusion of jequirity, develop and assimilate certain substances. acquiring a new physiological action, having the power of flourishing upon the conjunctiva of living animals and through the formation of a "ferment," produce certain reactions which we see in a well developed ophthalmia jequiritica. The action of ophthalmia jequiritica is similar to an acute blennorrhœa. The micrococcus that flourishes in and around the trachomatous bodies is suppressed by the more powerful bacilli of the jequirity infusion ; it is, "the survival of the fittest." With extinction of the jequirity ophthalmia, there is an atrophy of the original inflammatory products.

170 RANDOLPH STREET.

---

THE following advertisement is in a German medical paper: A miss of somewhat more than 30 years of age, but with a good-looking face; desires a bachelor-physician for her surgery, with which is connected a farm and a dry-goods store. He will be goodly fed, and he will obtain a good salary, if not more.

THE *Boletin de Ciencias Medicas* of Mexico, reports good results from the internal application of iodoform in diabetes insipidus. The iodoform is given in the form of pills with the extract of the tonka bean and conmarine, half a grain of the iodoform at a dose.

FIFTY cents will be paid by one of our subscribers for a single copy of this journal of the issue of December, 1881, to be left at room 37, No. 240 Wabash avenue.

COL. ROBERT MURRAY, Assistant Surgeon-General, and senior surgeon of the service, has been appointed by the President, Surgeon-General of the Army.

THE Cincinnati Directory for 1883 gives 463 as the number of physicians in that city.

